# Identification and screening of host proteins interacting with ORFV-ORF047 protein

**DOI:** 10.1186/s12985-021-01499-y

**Published:** 2021-01-26

**Authors:** Guohua Chen, Xiaobing He, Huaijie Jia, Yongxiang Fang, Xiaoxia Wang, Zhongzi Lou, Fan Yang, Weike Li, Zhizhong Jing

**Affiliations:** 1 State Key Laboratory of Veterinary Etiological Biology, Key Laboratory of Veterinary Public Health of Ministry of Agriculture, Lanzhou Veterinary Research Institute, Chinese Academy of Agricultural Sciences, Lanzhou, 730046 China; 2 School of Public Health, Lanzhou University, Lanzhou, 730046 China

**Keywords:** Protein–protein interactions, Yeast two hybrid, ORFV ORF047, Co-IP

## Abstract

**Background:**

Orf virus (ORFV) is a member of the genus *Parapoxvirus* and family Poxviridae. The virus has a worldwide distribution and infects sheep, goats, humans, and wild animals. However, due to the complex structure of the poxvirus, the underlying mechanism of the entry and infection by ORFV remains largely unknown. ORFV ORF047 encodes a protein named L1R. Poxviral L1R serves as the receptor-binding protein and blocks virus binding and entry independently of glycosaminoglycans (GAGs). The study aimed to identify the host interaction partners of ORFV ORF047.

**Methods:**

Yeast two-hybrid cDNA library of sheep testicular cells was applied to screen the host targets with ORF047 as the bait. ORF047 was cloned into a pBT3-N vector and expressed in the NMY51 yeast strain. Then, the expression of bait proteins was validated by Western blot analysis.

**Results:**

**S**heep SERP1and PABPC4 were identified as host target proteins of ORFV ORF047, and a Co-IP assay further verified their interaction.

**Conclusions:**

New host cell proteins SERP1and PABPC4 were found to interact with ORFV ORF047 and might involve viral mRNA translation and replication.

## Introduction

Poxviruses subvert host antiviral responses by their encoding proteins, which mimic, counteract, or interact with various cellular proteins. L1R protein is a myristoylated, 23–29 kDa transmembrane protein present on intracellular virions' outer membrane surface (INV) expressed during late infection [1, 2]. Poxviral L1R is required for viral entry and fusion with infected cells [3]. Previously, it was thought to be involved in virus assembly and morphogenesis [2], but later it was demonstrated that it is necessary for entry and vaccinia virus (VACV)-induced cell to cell fusion by associating with members of the entry-fusion complex, but not in assembly [4–6]. The monoclonal Ab (mAb) to L1R decreases VACV-induced cell to cell fusion at low pH, exhibits potent virus-neutralizing activity, and inhibits virus entry, but the specific role of L1R during viral entry is yet to be elucidated [4]. L1R serves as the receptor-binding protein for entry and blocks viral binding and entry independently of glycosaminoglycans[7]. The L1 protein is conserved in all poxvirus [8].

Orf virus (ORFV), a member of the genus *Parapoxvirus* of the *Poxviridae* family, causes contagious ecthyma. ORFV causes an infectious disease that commonly affects sheep, goats, and occasionally humans [9]. ORFV ORF047 is a 245 amino-acid (aa) myristoylated polypeptide with a predicted C-terminal transmembrane domain 183–204 aa, also named L1R, the construction similar to VACV L1. Until now, little is known about ORF047. The detailed role of ORF047 during ORFV entry and infection was not be elucidated yet. The best way to investigate the role of a protein is to find out its host interaction proteins. A yeast-two hybrid (YTH) was performed to screen and identify ORF047′s host target proteins against sheep testicular cellular cDNA library. YTH provide a powerful approach to identify protein-protein interaction; however, the split-ubiquitin membrane Yeast-two hybrid (MbYTH) system is a genetic technique to determine the interaction of membrane proteins [10–12], which utilizes complementation between the separable domain of ubiquitin to investigate membrane protein interactions. Several different laboratories have successfully applied the MbYTH system for proteins from a wide range of organisms. The MbYTH system has been used to investigate the interactions between plant sucrose transporters and between a natural resistance-associated macrophage protein and membrane-bound thioredoxin in Brassica juncea [13]. Furthermore, the MbYTH system has recently been used to isolate interacting partners of the human proteins, BAP31 and ErbB3, from human cDNA libraries [14]. As ORF047 encoding L1 protein is a membrane protein, the Split-ubiquitin cDNA libraries of sheep testicular cells were established to screen the host proteins interacting with ORF047.

As a result, three host proteins were identified. Further characterization of interactions between ORF047 and these proteins will contribute to understanding the function of ORF047 during the infection and entry.

## Material and methods

### Animal

Three of Newborn sheep lambs from Gan Su Agricultural-Farm Ranch Co, Ltd, which provided the testicular parenchyma was used for the preparation of primary sheep testicular cells.

### Virus and yeast strains and plasmids

ORFV strain (ORFV/QH01/2010) was isolated from the scar of a clinically ORFV infected sheep from Qinghai [15], was saved in the laboratory of Lanzhou Veterinary Research Institute (LVRI), China. Yeasts strains (NMY51), pBT3-N, pBT3-STE, PPRN-3, pTSu2-APP, pNubG-Fe65 plasmids (Table 1) for YTH experiments were obtained from Shanghai OE Biotech. Co. Ltd (Shanghai, China). 293 T cell, pcDNA-3.1–3×HA-N, and pcDNA-3.1–3×Flag-N plasmids (Table 1) are stocks in our lab. Cells were cultured using RPMI 1640 (Gibco, Grand Island, New York, USA) supplemented with 10% fetal calf serum (Gibco, Grand Island, New York, USA) and 100 mg/ml penicillin/streptomycin in a humidified 5% CO_2_ atmosphere at 37 °C.

**Table 1:** The table summarizing the general information about all the plasmids they used in this work

Plasmids name	The general information
pBT3-N	Bait vector used for constructing plasmid
pBT3-STE	Bait vector used for constructing plasmid
pBT3-N-ORF047	Recombinant Bait Plasmid
pPR3-N	Negative Control plasmid
pTSU2-APP	Positive Control plasmid
pOst1-NubI	Negative Control plasmid
pNubG-Fe65	Positive Control plasmid
pcDNA3.1–3×HA-N	Expression vector used for constructing expression plasmid
pcDNA3.1–3×Flag-N	Expression vector used for constructing expression plasmid
pcDNA-3.1–3×HA-ORF-047	Recombinant expression plasmid transfected cell for IP
pcDNA-3.1–3×Flag-FLC	Recombinant expression plasmid transfected cell for IP
pcDNA-3.1–3×Flag-SEPR1	Recombinant expression plasmid transfected cell for IP
pcDNA-3.1–3×Flag-PABPC4	Recombinant expression plasmid transfected cell for IP

### Construction of YTH cDNA library of sheep testicular cells

All trials were constructed with the approval of the Ethical Committee of the Lanzhou Veterinary Research Institute of the Chinese Academy of Agricultural Sciences. Neonatal sheep were fully anesthetized with isoflurane inhalation anesthetic administered by face mask (1 to 2% isoflurane) followed by exsanguination as an adjunctive method of euthanasia. These animals were placed in dorsal recumbency on the surgical table for placement of vascular introducer sheaths. Preparation of primary neonatal sheep testicular cells was performed as previously described [16] with few modifications. The testicular parenchyma by ophthalmic tweezers and scissors under sterile conditions. Cut the testicular parenchyma into tissue pieces of 1–2 mm size, and the small tissue pieces were then put into D-hanks solution and pipetted gently several times. After standing for 5–10 min, the supernatant was removed, and the pellet was allowed to digest with lysis buffer containing 0.1% IV collagenase (GIBCO) and 0.25% trypsin (GIBCO) at 4 °C for 12 h.

An equal volume of DMEM medium (HyClone) containing 10% fetal calf serum (GIBCO) was then added to stop the enzymatic digestions. After gentle pipetting several times using a Pasteur pipette, the tissue lysate was filtered with a 200-mesh copper wire screen. The digested solution was then collected and centrifuged at 1000 rpm for 10 min. The supernatant was discarded, and the pellet was washed twice with serum-free culture medium. The DMEM medium containing 10% fetal bovine serum was added to resuspend the cells. The cell viability was to determine by trypan blue staining. Then, the cells at a density of 1–2 × 10^5^ cells/mL placed in a 25 mm cell culture flask and were incubated at 37 °C in a humidified incubator with 5% CO_2_. After incubation, the culture media containing non-adherent cells were discarded. The DMEM culture medium containing 10% FBS was added to continue purifying the sheep testicular cells and subculture the cells for 5 passages. Finally, the cultural sheep testicular cells were collected and sent to Shanghai OE Biotech. Co. Ltd (Shanghai, China) for construction of the YTH cDNA library. The total RNA of sheep testicular cells was extracted and reverse transcribed into 1st strand cDNA. Following the normalization treatment and short fragment removal, the cDNA of sheep testicular cells was cloned into PPRN-3 vectors (Prey Plasmid).

### Bait plasmid construction

Previous, the ORF047 gene has been cloned from the DNA of ORFV, and the recombinant plasmid of pGEM- ORF047 was the stock in our laboratory [17]. Based on the characteristics of the bait vector, the specific primers (restriction site underlined): ORF047-F (5′-ATTAACAAG GC CATTACGGCCGGGGCCGCCGCCAGCATCCAGACCACC-3′), ORF047-R (5′- GACGGACG GCGGAAATTCCGTAAAGGGGCCGCCTCGGCCAATCAGTT-3′) was designed. Subsequently, the PCR product was cloned and digested with restriction enzyme SfiI (NEB, USA) and inserted into a pBT3-N plasmid. The recombinant pBT3-N-ORF047 plasmid (bait plasmid) was confirmed by restriction enzyme digestion and sequencing (Sangon Biotech, Shanghai, China).

### Bait plasmid expression in yeast cells

Following the manufacture's protocols of the Yeast maker™ Yeast Transformation System 2 kit (Cat. No.630439, Clontech, USA), the transformants of pSTU2-APP plasmids were used as a positive control. The pPR3-N plasmid was used as a negative control. The pBT3-N-ORF047, pSTU2-APP, pPR3-N plasmids were transformed into NMY51, respectively. Transformants were grown on SD/-Leu, SD/-Trp agar plates at 30 °C for 3–5 d. To check the ORF047 bait plasmid expression in NMY51, one colony from the SD/-Trp plate was inoculated into SD/-Trp broth and grown to 0.6 OD600 at 30 °C, 250 rpm. The total proteins were subsequently extracted from the centrifuged pelleted cells by the Y-PER yeast protein extraction reagent (Thermo, RF-236781). The extracted proteins were separated by 12% SDS-PAGE and electro-blotted onto PVDF membrane (Millipore, Billerica, Massachusetts, USA) for western blot analysis. The ORF047 bait expression was detected with anti-Lex A mouse McAb (Cat. No.SC-390386, Santa Cruz, USA), followed by m-IgGk BP-HRP second antibody (Cat. No.sc-516102 Santa Cruz, USA), with positive signals revealed, all the membranes were imaged using the ChemiDoc XRS + system (Bio-Rad).

### The dual membrane function assay of bait plasmid

Following the manufacture's protocols of the YTH System (Clontech, Mountain View, California, USA), the pBT3-N-ORF047 and pOst1-NubI, pBT3-N-ORF047 and pPR3-N, pSTU2-APP and pNubG-Fe65, pSTU2-APP and pPR3-N plasmids were transformed into NMY51, respectively. Transformants were grown on SD/-Trp-Leu, SD/-Trp-Leu-His, SD/-Trp-Leu-His-Ade agar plates at 30 °C for 4 d. Count the number of colonies on all plates (SD/-Trp-Leu-His and SD/-Trp-Leu-His-Ade) versus nonselective plates SD/-Trp-Leu. Suppose pBT3-N-ORF047 bait is properly expressed and functional in the DUALmembrane functional assay. In that case, we should observe between 10 and 100% growth on SD/-Trp-leu-his and SD/-Trp-leu-his-ade plates derived from transformation reaction of pBT3-N-ORF047 and pOst1-NubI plasmids, depending on the expression level of your bait growth under selection indicates that your bait is well expressed and that the Cub moiety is accessible for interaction with the Ost1-NubI moiety expressed from the pOst-NubI control prey. It should observe no significant growth on selective plates derived from the transformation reaction of pBT3-N-ORF047 and pPR3-N plasmids, as bait does not interact strongly with the NubG fused nonsense-peptide expressed from the pPR3-N control prey. Transformation reaction of pSTU2-APP and pNubG-Fe65 should yield robust growth under selection since the APP bait (pSTU2-APP) is well expressed and interacts strongly with Fe65 prey fusion. Transformation reaction of pSTU2-APP and pPR3-N (the negative control) should yield considerably fewer colonies than a reaction of pSTU2-APP and pNubG-Fe65. If the bait interacts with the Ost1-NubI control prey but not with the pPR3-N derived NubG-nonsense-peptide prey, the bait plasmid could be used in the YTH screening.

### YTH screening by co-transformation of bait with prey plasmids

To screen the host proteins to interact with ORF-047 bait against a NubG-fused cDNA library of sheep testicular cells, pBT3-N-ORF047 and prey plasmids were co-transformed into NMY51 with Yeastmaker™ Yeast Transformation System according to the DUALmembrane starter kits User Manual (Dualsystems biotech). The pBT3-N-ORF047 and prey plasmids were co-transformed into NMY51, and the transformants were then grown on SD/-Trp-Leu, SD/-Trp-Leu-His, SD/-Trp-Leu-His-Ade/X-Gal agar plates at 30 °C for 3–5 d. Blue colonies were patched out onto higher stringency SD/-Trp-Leu-His-Ade/X-Gal agar plates. Primers of pPR3-N-F/R were applied to amplify each insert DNA of potential positive prey plasmids.

### Confirmation of the interactions

pBT3-N-ORF047 bait was co-transformed into NMY51 with each prey plasmid in putatively positive hits to confirm the interactions. The prey plasmids were briefly extracted from putatively positive clones using the Easy Yeast Plasmid Isolation Kit (Cat. No. 630467, Clontech, Mountain View, California, USA). Subsequently, each prey plasmid was transformed into *E. coli DH5α* competent cells (Transgen, Beijing, China), and purified from transformants growing on selected LB/Amp agar plates using the Plasmid MiniKit I (Cat. No. D6943-02, Omega, Doraville, Georgia, USA). Following this, each putatively positive prey plasmid was co-transformed with pBT3-N-ORF047 bait and pBT3-N plasmids into NMY51 and the co-transformants grown on SD/-Trp-Leu-His-Ade/X-Gal plates to test for interactions. Co-transformant containing pSTU2-APP and pNubG-Fe65, grown on SD/-Trp-Leu-His-Ade /X-Gal, was used as a positive control, and co-transformants containing pSTU2-APP and pPR3-N, grown on SD/-Trp-Leu-His-Ade /X-Gal, was used as a negative control. Blue colonies indicated true positive interactions under these conditions. To verify positive clones, the prey plasmids were sequenced using the pBT3-N primers, and the sequencing results were analyzed by blasted in NCBI.

### Positive prey analysis

The sequencing results were analyzed by blasted in NCBI, which revealed that three inserts had a 100% sequence identity to that of the three Ovis aries genes: databases to analyze the corresponding function.

### Construction of PABPC1, SERP1, FLC, ORF047 expression plasmid

The expression primers were designed based on the published sequences: SERP1 gene (XP_014948090.1), the forward primer, 5′AAGCTTATGGTCGCCAAGCAGCGGA 3′(the underlined sequence is the *HindIII* site) and the reverse primer,5′AATGAATTCTCACATGCCCATCCTGATAC -3′ (the underlined sequence is the *EcoR I* site). FLC gene(XP_014961714.1) AAGCTTATGAGCTCCCAGATTCGTCAG(the underlined sequence is the *HindIII* site) and the reverse primer, 5′ GAATTCCTAGTCGTGCTTGAGGGT3′ (the underlined sequence is the *EcoR I* site). PABPC4 gene (XM_004001826.3), The forward primer, 5′AAGCTTATGAACGCTGCGGCCAGCAGCTAC3′ (the underlined sequence is the *Hind III* site), 5′GCGGCCGCCTAAGAGGTAGCAGCAGCAAC3′ (the underlined sequence is the *Not I* site).

Sheep testicular cells were collected and adjusted to 1 × 10^7^/mL, and the cells were washed with PBS by centrifugation at 2000 g for 7 min. Total RNA was extracted from the collected cells with the Catrimox-14TM RNA kit (TaKaRa Corporation, China). cDNA was synthesized at 42 ℃ using oligo (dT)-adaptor primer and avian myeloblastosis virus (AMV) reverse transcriptase. The complete sequence of PABPC1, SERP1, or FLC was amplified from synthesized cDNA based on the product were analyzed by electrophoresis on a 1% agarose gel. The fragment gene with a length of 201 bp, 528 bp, 1983 bp separately was subcloned into a pCDNA3.1–3× Flag-N expression vector (Invitrogen, USA). ORF047 gene was subcloned into a pCDNA3.1–3×HA-N expression vector. The mutation-free recombination plasmids were confirmed by sequencing and subsequently transformed into JM109 Escherichia coli cells. Recombinant plasmids were selected by blue-white selection (Takara Biotechnology).

### Western blot and co-immunoprecipitation assay

To detect the interaction between ORF047 bait and prey proteins, HEK293T cells were transfected with pcDNA-3.1-HA-ORF047 and cotransfected with either pcDNA-3.1–3× Flag-FLC or pcDNA-3.1–3× Flag-SERP1 pcDNA-3.1–3× Flag-PABPC4 plasmids for the Co-IP assay. Cells were harvested and washed two times with cold phosphate-buffered saline (PBS), then pretreated with 0.5 ml NP-40 lysis buffer ( Beyotime, P0013F) with a cocktail of many protease inhibitors (1:1000) centrifuged at 12 000 rpm for 15 min at 4 °C, and the supernatants were collected. For immunoprecipitation, 0.4 ml of cell lysate was incubated with 1.5 mg beads coated with anti- FLAG antibody (Sigma) for ≥ 60 min on a rotator at 4 °C, then the tubes were placed on the magnet for 1 min and remove the supernatant, and the beads were washed 3 times in 500 µl pre-chilled lysis buffer, and resuspended in 50 µl 1 × Laemmli (loading) buffer and boil for 5 min at 95 °C, the tubes were then put on the magnet for 1 min, and the supernatant was transferred to fresh tubes. The proteins were resolved by electrophoresis on 12% Bis-Tris polyacrylamide gels (Shanghai Sangon Biotech, China) and transferred to polyvinylidene fluoride membranes (Immobilon-P Transfer membranes, Millipore). Membranes were blocked for 12 h at 4 °C in 5% (wt/vol) Tris-buffered saline supplemented with 0.1% Tween 20 (TBST)-diluted milk (BSA, Amresco) buffer. Membranes were incubated with primary antibody diluted in 5% (wt/vol) BSA and 1 × TBST at room temperature. The primary antibodies used include anti-HA (66006-1, Proteintech), anti-FLAG (F3165, Sigma-Aldrich). The blots were detected by the enhanced chemiluminescence detection kit (#1705062, Bio-Rad) after incubation with an appropriate secondary antibody conjugated to horseradish peroxidase. All the membranes were imaged with the ChemiDoc XRS + system (Bio-Rad).

## Results

### Construction of YTH cDNA library of sheep testicular

**Fig. 1:**
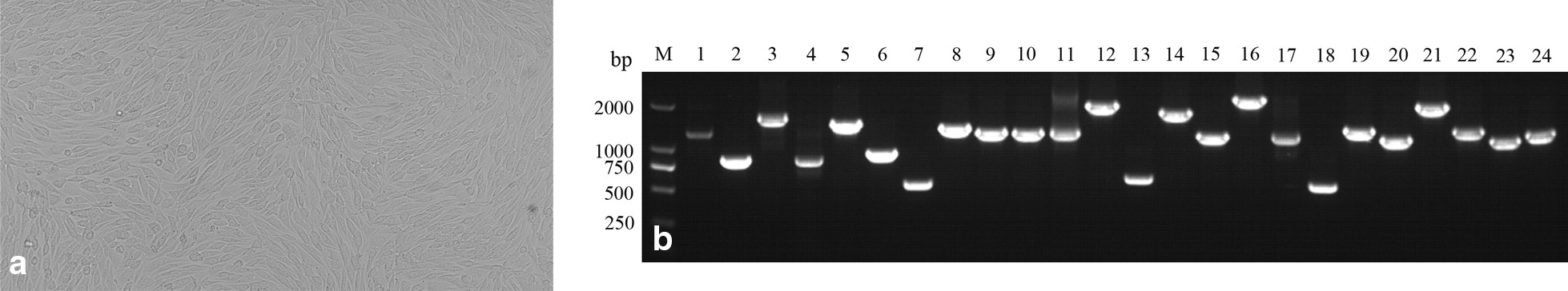
**A** Sheep testicular cells after 6 generations of culture. **B** Identification of the inserted DNAs from the cDNA library. Gel electrophoresis of the inserted DNAs. Lanes 1–24 were 24 individual recombinant colonies, randomly picked and amplified by performing PCR with the PPR-N vector universal primers. All of the fragments were more than 250 bp. 14 samples were about 1000 bp, 3 samples were about 500 bp

Sheep testicular cells were separated and cultured. When the cells were cultured 5–6 generations (Fig. 1a), they were collected. 5 × 10^7^ cells were obtained and sent to Shanghai OE Biotech. Co. Ltd for cDNA library construction. As a result, the cDNA library titer was 7 × 10^5^ CFU and the inserted DNAs were from 200 to 2000 bp according to the sequencing results of 40 clones (Fig. 1b). These results indicated that the cDNA library could be used for YTH screening. Besides, the prey plasmid provided by OE Biotech Co. Ltd was 1 mg/ml.

### Construction of pBT3-N-ORF047 bait plasmid and ORF047 expression plasmid

**Fig. 2:**
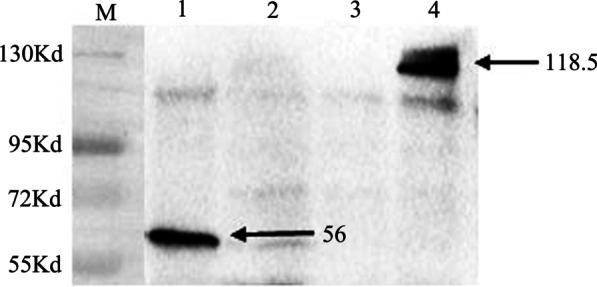
Western blot analysis of expression of the ORF047 Bait Plasmid in NMY51. Transform pBT3-N-ORF047 plasmids into the yeast strain NMY51 to verify expression by Western blot. Lane 1. The protein expression of NMY51 transform pBT3-N-ORF047 plasmid; Lane 2,3. The protein expression of NMY51 transforms negative control pPR3-N plasmid; Lane 4. The protein expression of NMY51 transforms positive control, pTSU2-APP plasmid

The ORF047 gene fragment of 735 bp was amplified from a pGEM-ORF047 plasmid [17], then pcDNA-3.1–3×HA-ORF047 expression plasmid and pBT3-N-ORF047 bait plasmid were constructed. The construction of two recombinant plasmids was confirmed by restriction enzyme digestion analysis. The bait plasmid is co-transformed with the control plasmids pTSU2-NubI and pPR3-N into NMY51. If white colonies were grown on SD/-Leu and SD/-Trp plates after 4 d culture, the bait was confirmed with no toxicity. To detect the fusion proteins' expression, each transformant is inoculated in a liquid selective medium and grown overnight. Total proteins of the NMY51 transformed with bait plasmid were extracted and detected by Western blot with anti-Lex A mouse mAb. The result showed that the negative control (NMY51 transformed with pPR3-N) did not yield the detected band; the bait was properly expressed and yielded a band of the molecular weight of 28 kDa plus 38 kDs for the Cub-LexA-VP16 fusion, which was consistent with its predicted size (Fig. 2). The positive control of pSTU2-APP plasmid transformed NMY51 yielded a band of a molecular weight of 118.5 kDa (Fig. 2).

### The DUALmembrane function assay of the bait plasmid

**Fig. 3:**
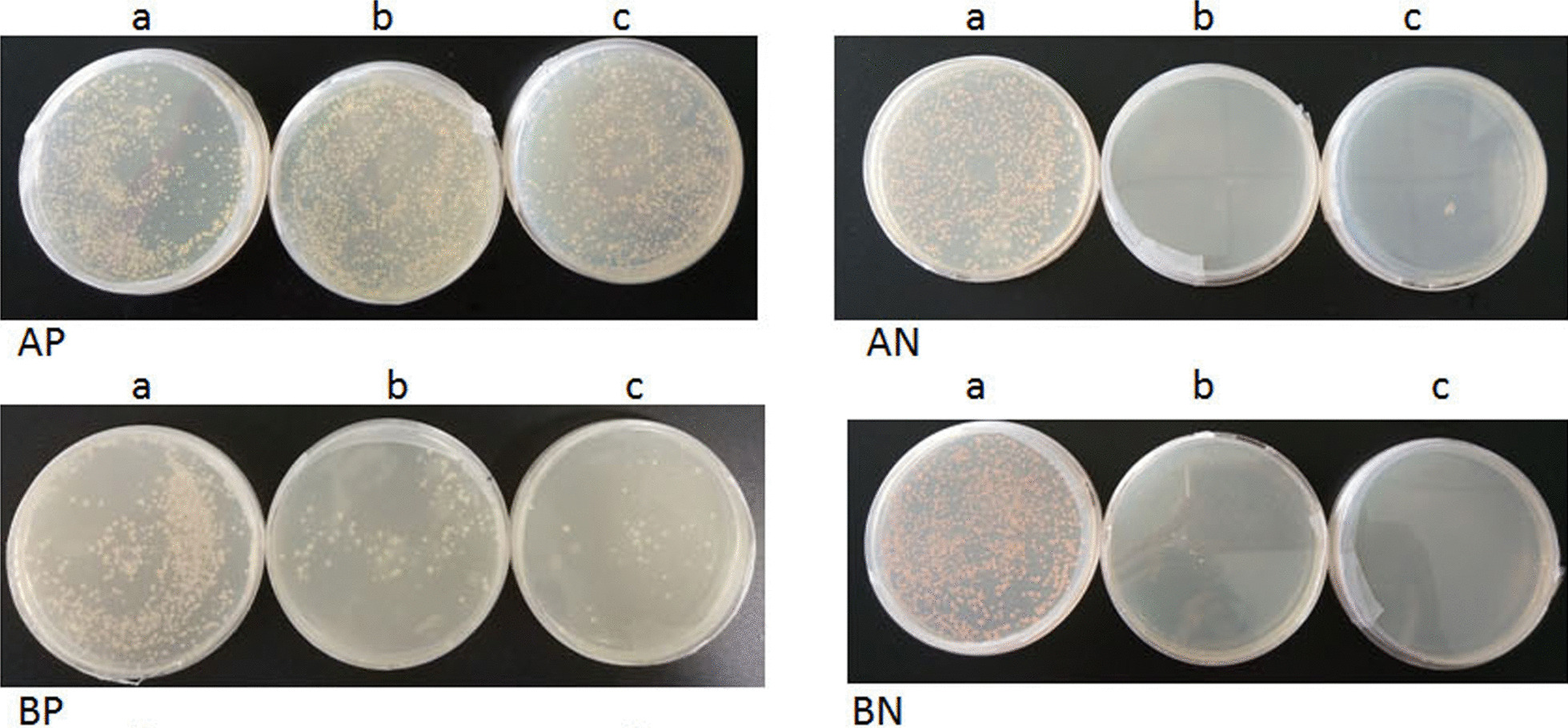
Detection of bait vectors auto-activation and toxicity. AP: The positive control pTSU2-APP + pNubG-Fe65; AN: The negative control pTSU2-APP + pPR3-N; BP: pBT3-N-ORF047 + pOst1-NubI; BN: pBT3-N-ORF047 + pPR3-N; **a** SD/-Leu/-Trp medium; **b** SD/-His/-Leu/-Trp medium; **c** SD/-Ade/-His/-Leu/-Trp medium

The positive plasmid (Ost1-NubI) were transformed into NMY51, and subsequently, the transformants were grown on SD/-Trp-Leu, SD/-Trp-Leu-His, SD/-Trp-Leu-His-Ade plates. Co-expression of bait with Ost1-NubI should result in split-ubiquitin's reconstitution through the strong affinity of wild type Nub for Cub and the concurrent activation of reporter genes. Growth of yeast expressing bait and the Ost1-NubI control signifies that the bait is functional in the DUALmembrane system. However, the negative control plasmid (pPR3-N prey plasmid) and pBT3-N-ORF047 bait plasmid were transformed into NMY51, but co-expression of bait with the NubG-nonsense peptide fusion expressed from the pPR3-N prey vector should not lead to split ubiquitin formation, as NubG has no affinity for Cub, bait is unlikely to interact with the nonsense peptide fused to NubG. Consequently, yeast coexpressing bait and the NubG-nonsense peptide fusion did not grow on selective medium as positive control transformants, co-expression of Cub-fused APP and NubG-fused Fe65 from pTSU2-APP and pNubG- Fe65 should lead to robust growth on selective medium as APP and Fe65 interact with each other and consequently initiate the split ubiquitin mechanism, which confirmed that pBT3-N-ORF047 bait plasmid is functional in the DUALmembrane assay and fit for the conditions of a library screen (Fig. 3).

### Sequencing and analysis of positive prey

**Fig. 4:**
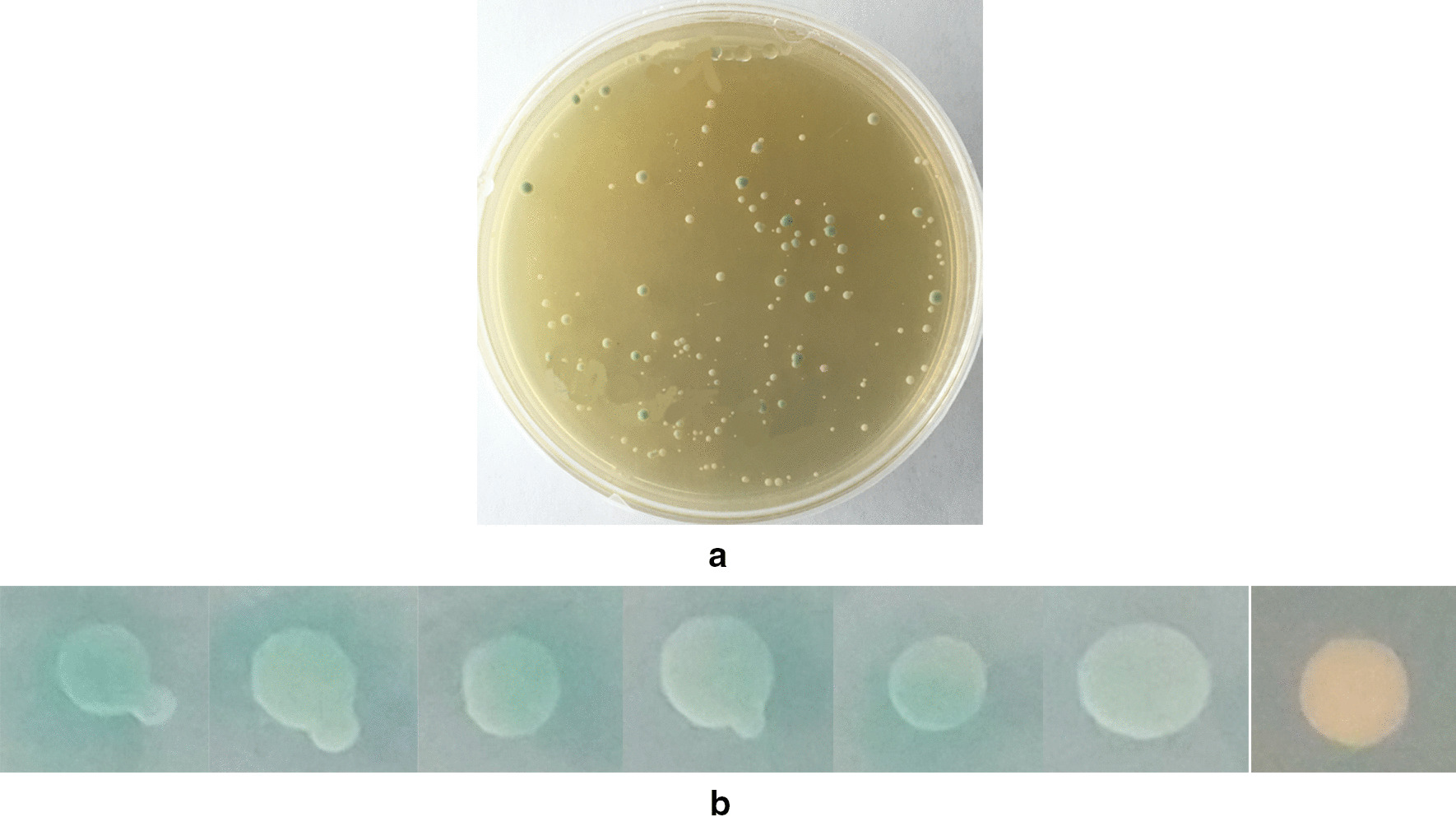
The analysis of putative colonies of cDNA library plasmid and bait vector yeast two hybrids. **a** Yeast clones are grown on SD/-Ade/-His/-Leu/-Trp + xgal; **b** Detection of β-galactosidase activity of the positive yeast clones

A total of 30 colonies were grown on selective SD/-Trp-Leu-His agar plates (Fig. 4a). Six blue colonies were grown on SD/-Trp-Leu-His-Ade/X-Gal agar plates (Fig. 4b). To verify the nucleotide sequence of the identified host proteins interacting with pBT3-N-ORF047, blue colonies were inoculated into SD/Leu-Trp broth and grown to 0.6 OD600 at 30 °C. A total of 6 pPR3-cDNA positive plasmids were patched out, extract the yeast plasmid, transformed into Escherichia coli, sequenced using the primers of pPR3-N-F/R. One-to-one mutual rotation verification, and sequence to get 3 positive gene The sequences were analyzed using the BLAST. The three fragments had 100% similarity with Ovis aries stress-associated endoplasmic reticulum protein 1 (SERP1) (XP_014948090.1), musimon ferritin light chain (FLC) (XP_011998831.1), polyadenylate-binding protein 4 isoform X1 (PABPC4) (XP_004001875.1) (Table 2). The sequences encoded 67, 176, and 661 aa of SERP1, FLC, and PABPC4.

**Table 2:** The table summarizing the results of sequencing analyzes results

Gene number	Gene name	Gene bank	ORF true or not
C1	XP_014948090.1	PREDICTED: stress-associated endoplasmic reticulum protein 1 [Ovis aries]	True
C2	XP_011998831.1	PREDICTED: ferritin light chain [Ovis aries musimon]	True
C4	XP_004001875.1	PREDICTED: polyadenylate-binding protein 4 isoform X1 [Ovis aries]	True
C5	XP_012030026.1	PREDICTED: ubiquitin carboxyl-terminal hydrolase 15 isoform X5 [Ovis aries]	Not
C6	XP_011989158.1	PREDICTED: alpha-enolase isoform X4 [Ovis aries musimon]	Not
C7	XP_011987886.1	PREDICTED: nestin isoform X1 [Ovis aries musimon]	Not

### PABPC4, SERP1, and FLC physically interact with ORF047

**Fig. 5:**
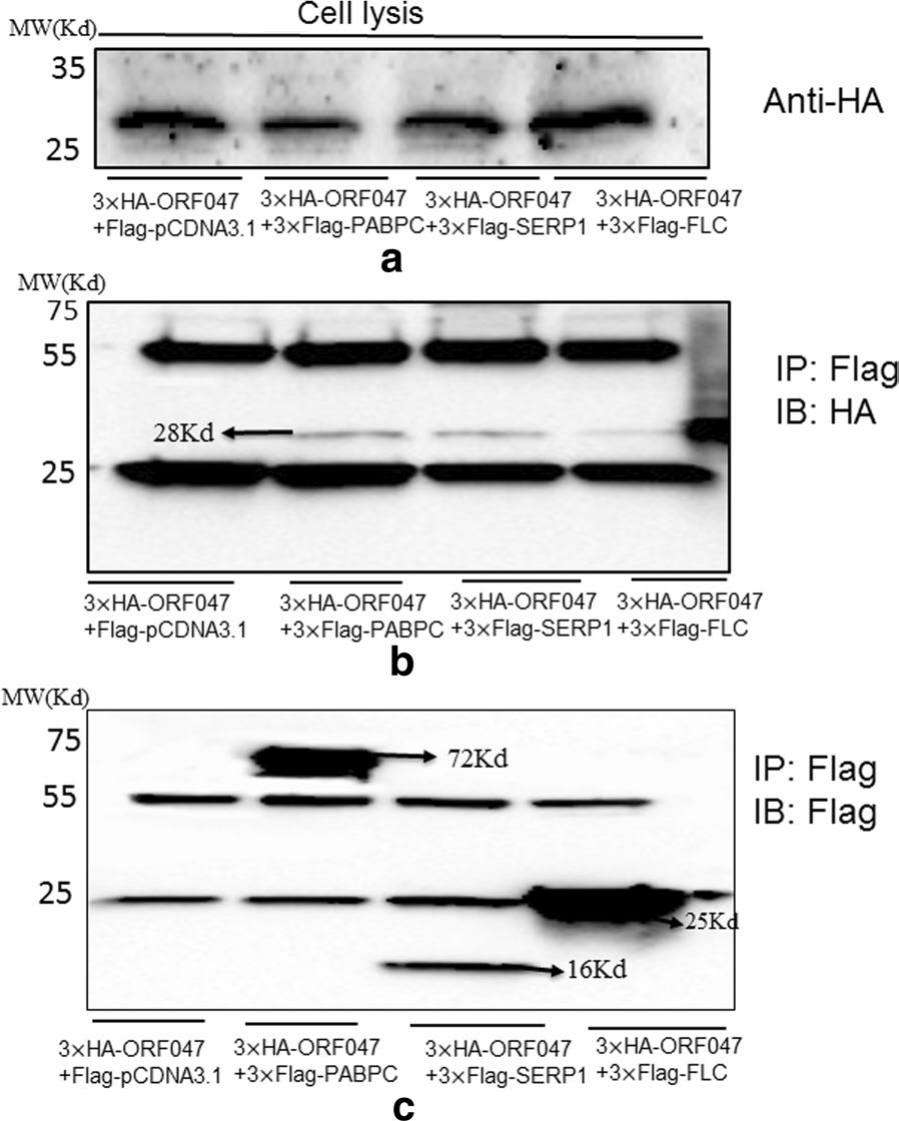
ORF047 interacts with PABPC1 or FLC and SERP1. 293 T cells were transfected with different plasmid groups(pcDNA-3.1–3×HA-ORF047and pcDNA-3.1–3×Flag plasmids or pcDNA-3.1–3×HA-ORF047 and pcDNA-3.1–3×Flag-PABPC4 plasmids or O pcDNA-3.1–3×HA- ORF047 and pcDNA-3.1–3×Flag-PABPC4 plasmids or pcDNA-3.1–3×HA-ORF047 and pcDNA-3.1–3×Flag-SEPR1 plasmids) for immunoprecipitation. Immunoprecipitation analysis was carried out 48 h later using anti-HA monoclonal antibodies and anti-Flag antibodies. **A** Western blot analysis cells lysis transfected with different plasmid groups with anti-HA monoclonal antibodies, the results showed pcDNA-3.1–3×HA-ORF047 protein expression in 293 T cells lysis using anti-HA monoclonal antibodies **B** Western blot analyzed immunoprecipitation with anti-Flag monoclonal antibodies and immunoblotting with anti-HA monoclonal antibodies. The results showed the interaction between ORF047 with the PABPC4 protein or SERP1 or FLC protein. **C** Western blot analyzed immunoprecipitation and immunoblotting with anti-Flag monoclonal antibodies

Physical interactions between ORF047 and PABPC4, SERP1, or FLC were examined by the Co-IP assay using anti-HA antibodies and immunoblotting with anti-Flag antibodies. The expression of ORF047-HA was detected as expected (Fig. 5A). Co-IP was performed with HA mAb coated beads, and the elute was detected with HA and Flag mAb, respectively. As a result, PABPC4, SERP1, and FLC could be pulled down by HA tag (Fig. 5B, C), indicating a physical interaction between ORF047 and PABPC4, SERP1, and FLC.

## Discussion

ORF047 was chosen in this study based on the biological properties of the L1 protein, the most investigated viral protein of the poxviridae family, vaccinia virus (VACV). VACV L1 is synthesized during late infection and is a myristylated component of the INV membrane [18]. Blocking L1 expression aborts morphogenesis and reduces the incorporation of DNA into virions [7]. L1 plays an important role during viral entry; however, a study has suggested that VACV L1 functions as a receptor binding proteins by engaging host receptors unique from GAGs [4].

Comparative analysis of L1R protein sequences of animal poxviruses showed that a highly conserved myristoylation motif (G-X-X-X-S)) and six conserved cysteine residues present in all poxviruses [8]. ORF047 which shows tertiary structure similarities to members of the VACV by analysis of tertiary structure homology modeling. The function of ORFV-L1 during entry is unknown, and its vaccine is lacking. In this study, we investigated host proteins that interact with the L1 proteins of ORFV. Several powerful techniques have been developed for identifying and confirming interaction proteins. The YTH screen system is a powerful tool for high-throughput screening of unknown interaction partners [12, 13]. However, a YTH screen always yields a varying number of false-positive candidates, which need to be confirmed by biologically relevant cells' techniques. The Co-IP assay was a powerful technology for further verification of YTH results.

In this study, the aa sequence analysis of ORF047 revealed that ORF047 possess putative transmembrane domains and encodes a membrane protein (data not shown). The split-ubiquitin membrane YTH utilizes complementation between separable ubiquitin domains to study membrane protein interaction [13]. Therefore, the split-ubiquitin membrane cDNA library of sheep was constructed. Three interaction proteins of ORF047, PABPC4, and SERP1and FLC were screened and verified by YIH and Co-IP. There is probably other host protein that interacts with ORF047, but it was not detected in our screening, probably because the cDNA library may not contain all the clones.

PABPCs family have five members (PABPC1, PABPC2, PABPC3, PABPC4, PABPC5), collectively refer to PABP, although it is unclear to what extent the functions and expression of these proteins overlap and previous studies have shown that PABPCs play important roles in translation, control the rate of mRNA deadenylation, and participate in mRNA decay [19]. PABPC1 and PABPC4, initially described as inducible poly(A) binding protein, is a homology of PABPC expressed in most cell types [20, 21]. PABPC4 has 75% homology with PABPC1 at the protein level and has similar binding poly(A) affinity with PABPC1. Many studies have shown that PABPC1 is its common cellular target to RNA virus or DNA virus [22]. Moreover, PABPC1 plays an important role in the infection and replication of the influenza virus [23], porcine reproductive and respiratory syndrome virus [24], and Rift Valley fever virus. Therefore, we speculated the interaction between PABPC4 and ORF047 might have an important effect on ORFV infection and replication.

SERP1, also known as ribosome-associated membrane protein 4, demonstrates a role in controlling membrane protein biogenesis at the ER [25, 26]. Previous research has shown SERP1 involved in many cellular processes, causing the accumulation of unfolded proteins and regulating cell apoptosis and the nuclear factor-κB signaling pathway. But, the role of interaction between SERP1 and ORF047 on the ORFV is needed to research. In a word, the interaction between PABPC4, SERP1, and ORFV ORF047 will provide several pieces of information on ORFV infection and replication.

## Conclusion

In this study, ORF047 was used as bait to screen its interacting host protein from the cDNA library of sheep testicular cells, and three host proteins PABPC4, SERP1 and FLC, were screened and verified. It was for the first time that PABPC4 and SERP1 were found to interact with ORF047.

## Data Availability

Data and materials are available upon request from the corresponding author.

## Abbreviations

ORFV: Orf virus
GAGs: Glycosaminoglycans
YTH: Yeast two-hybrid
INV: Intracellular virions
MbYTH: Membrane Yeast-two hybrid
Co-IP: Co-immunoprecipitation
VACV: Vaccinia virus
PABPC4: Polyadenylate-binding protein 4 isoform X1
SERP1: Stress-associated endoplasmic reticulum protein 1
FLC: Ferritin light chain
